# Impact of mRNA-Assessed Molecular Subtype Conversion, Intact and Apoptotic Circulating Tumor Cells on Survival of Metastatic Breast Cancer Patients: Proof of Principle

**DOI:** 10.3390/diagnostics10060369

**Published:** 2020-06-04

**Authors:** Stefan Stefanovic, Thomas M. Deutsch, Ralph Wirtz, Andreas Hartkopf, Peter Sinn, Maximilian Kohler, Jan Hofmann, Sanja Bankovic, Katja Vassilev, Marc Sütterlin, Andreas Schneeweiss, Markus Wallwiener

**Affiliations:** 1 Department of Gynecology and Obstetrics, Mannheim University Hospital, University of Heidelberg, Theodor-Kutzer-Ufer 1-3, 68167 Mannheim, Germany; maximilian.kohler@umm.de (M.K.); jan.hofmann@umm.de (J.H.); katja.vassilev@umm.de (K.V.); marc.suetterlin@umm.de (M.S.); 2 Department of Gynecology and Obstetrics, Heidelberg University Hospital, Im Neuenheimer Feld 440, 69120 Heidelberg, Germany; thomas.deutsch@med.uni-heidelberg.de (T.M.D.); markus.wallwiener@med.uni-heidelberg.de (M.W.); 3 Stratifyer Molecular Pathology GmbH, Werthmannstr. 1c, 50935 Cologne, Germany; ralph.wirtz@stratifyer.de; 4 Department of Women’s Health, University Hospital Tübingen, Calwerstr. 7, 72076 Tübingen, Germany; andreas.hartkopf@med.uni-tuebingen.de; 5 Department of Pathology, Heidelberg University Hospital, Im Neuenheimer Feld 224, 69120 Heidelberg, Germany; peter.sinn@med.uni-heidelberg.de; 6 Department of Hematology and Immunology, Clinical Center of Nis, University of Nis, Bulevar Zorana Djindjica 48, 18000 Nis, Serbia; sanja.bankovic3@gmail.com; 7 National Center for Tumor Diseases (NCT) Heidelberg, Im Neuenheimer Feld 460, 69120 Heidelberg, Germany; andreas.schneeweiss@med.uni-heidelberg.de; 8 German Cancer Research Center (DKFZ), Heidelberg, Im Neuenheimer Feld 280, 69120 Heidelberg, Germany

**Keywords:** breast cancer, circulating tumor cells, intrinsic subtype, biomarker conversion, survival, RT-qPCR

## Abstract

Breast cancers (BC) can mutate, allowing metastatic tumors (MT) to sometimes differ to primary tumors (PT) in gene expression. Despite contemporary metastatic breast cancer (MBC) therapy, subtype conversion seems prognostically disadvantageous. We strived to determine the influence of mRNA-assessed intrinsic subtype stability comparing PT and MT biopsies and circulating tumor cell (CTC)-based liquid biopsies on progression free survival (PFS) and overall survival (OS). Additional analyzed prognostic factors were PT subtype, MT subtype and hormone receptor loss. Kaplan-Meier curves and the log rank tests were used to compare PFSs and OSs. The proportions of luminal B and triple negative subtype MTs were increased compared to those observed in PTs. Fifteen patients were found to have tumors that underwent intrinsic subtype conversion and their OS was significantly decreased (*p* = 0.038). No such difference was observed when it comes to PFS. The majority of these tumors switched to a more aggressive intrinsic subtype. No significant differences in PFSs or OSs were observed between subtype converters with triple negative PTs compared to those with luminal subtype PTs. The same is true of subtype stable patients. Total CTC, iCTC and aCTC counts decreased with therapy, but there were no significant differences between subtype converters and subtype stable patients. Our data confirm a poorer overall survival of the intrinsic subtype converters and emphasize the importance of acquiring biopsies and re-biopsies of all available metastatic lesions alongside with CTC-based liquid biopsies for earlier recognition of patients with poorer prognosis and in need of altered individualized therapy regimens.

## 1. Introduction

Breast cancer (BC) remains an important cause of morbidity and mortality in women worldwide [1]. Metastatic breast cancer (MBC) is still associated with a dismal prognosis despite numerous technological advances and ongoing research efforts on a global scale [2]. In an era of individualized medicine, numerous targeted therapeutic regimens have been instituted and are being employed on a daily basis [3,4]. Furthermore, distinct BC intrinsic subtypes were defined in order to better guide therapy [5,6]. Triple negative tumors in particular, due to the lack of adequate drug targets, were shown to be associated with an unfavorable prognosis [7,8]. The same statement used to be true for any BC expressing HER2 prior to the introduction of various HER2-targeted therapies [7,9,10].

It has been recognized that a BC can, during its natural history, change its molecular subtype and consequently downregulate or change its receptor expression making it a proverbial moving target [11,12,13,14]. Biopsies of metastatic tumor (MT) tissues have confirmed suspicions that MTs can indeed have a genetic makeup different to that of the primary tumor (PT). That being said, MBC therapy is currently dependent on the MT’s biomarker profile [15]. However, some authors have posited that despite modern therapeutic modalities, intrinsic subtype conversion remains disadvantageous from a prognostic perspective [7,10,14,16]. Furthermore, the question remains whether all such conversions are equally disadvantageous [17,18].

Circulating tumor cells (CTCs) can be detected in peripheral venous blood samples in patients with MBC and sampling of such cells could possibly serve as a surrogate for a biopsy of the MT and this is why some authors have dubbed CTC enumeration a “liquid biopsy”. In our previous study, we were able to show that both patients with luminal and triple negative tumors had a significant downregulation of apoptotic circulating tumor cells (aCTCs) in their sera [19].

In this sequel study, our goal was to determine the influence of RT-qPCR based intrinsic subtype conversion on progression free survival (PFS) and overall survival (OS) and compare it to the impact of intact and apoptotic circulating tumor cells on survival. In addition, we evaluated the association between particular primary tumor intrinsic subtypes and PFS and OS in both the intrinsic subtype stable patients and the subtype converters. In addition, we analyzed the differences between subtype converters and subtype stable patients in regard to total CTC, iCTC and aCTC counts both at baseline and after specific therapy.

## 2. Results

Thirty-four patients fulfilled the biologically and methodologically highly demanding enrollment criteria in the analyzed timeframe. The mean age at the time of PT biopsy was 52.3 ± 9.7 years, while the age at which a metastatic focus had been diagnosed and biopsied was 58.1 ± 10.6 years. None of the patients in our cohort had a Grade 1 tumor, while G2 tumors were most common at 53% (Table 1). Luminal A and luminal B subtype PTs were both found in 38.2% of patients, while 20.6% had a triple negative PT subtype. Compared to PTs, higher frequencies of luminal B and triple negative MTs and a lower frequency of luminal A MTs were observed (Table 1). None of the patients had a PT or MT that was of the HER2 enriched subtype. Fifteen patients (44.1%) had tumors that underwent intrinsic subtype conversion.

**Figure 1:**
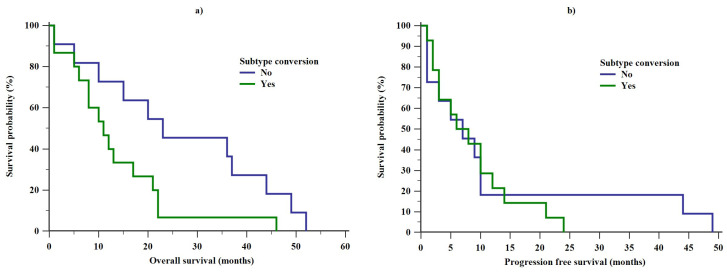
Differences in (**a**) overall survival (OS) and (**b**) progression free survival (PFS) between subtype stable patients and subtype converters.

OS was found to be significantly decreased (log rank test, Chi square = 4.3, *p* = 0.038) in patients whose tumors had undergone subtype conversion (Figure 1). The median OS for subtype stable patients was 23 months (range: 1–52) compared to 11 months (range: 1–46) in subtype converters. However, the stability of a BC’s intrinsic subtype was not associated with a significant difference in PFS (log rank test, Chi square = 0.171, *p* = 0.68).

**Figure 2:**
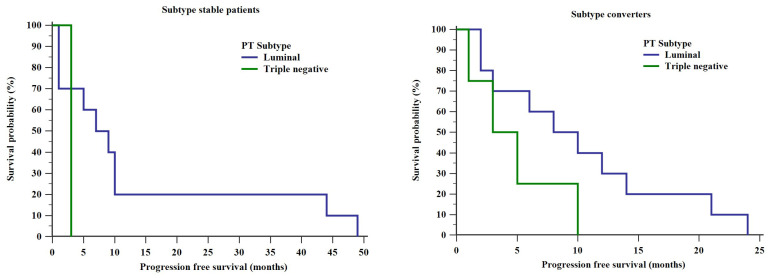
Differences in PFSs between different primary tumors (PT) subtypes in subtype stable patients and subtype converters.

PFS was shorter in subtype converters with triple negative PTs compared to those with luminal subtype PTs—a median of 4 months (range: 1–10 months) compared to 9 months (range: 2–24 months), respectively. This difference proved not to be statistically significant (log rank test, Chi square = 2.345, *p* = 0.126) (Figure 2). Data on only a single patient with a triple negative subtype stable BC were available. Hence, statistical analysis of the curves depicted on the left in Figure 2 would not be useful.

**Figure 3:**
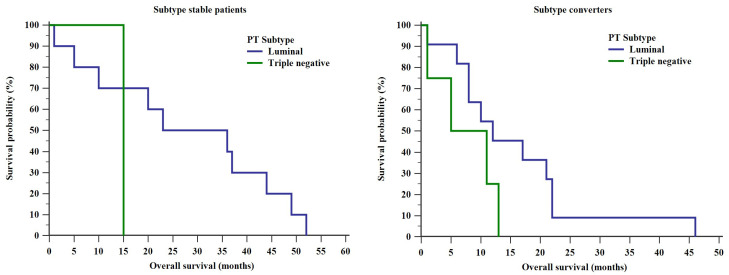
Differences in OS between different PT subtypes in subtype stable patients and in subtype converters.

OS was also shorter in subtype converters with triple negative PTs when compared to luminal type PTs—a median of 8 months (range: 1–13 months) compared to 12 months (range: 1–46 months), respectively (Figure 3). However, analysis of the Kaplan–Meier curves did not demonstrate a statistically significant difference (log rank test, Chi square = 2.359, *p* = 0.125). A single subtype stable triple negative patient was identified limiting a statistical analysis of OS curves in the left pane of Figure 3.

**Figure 4:**
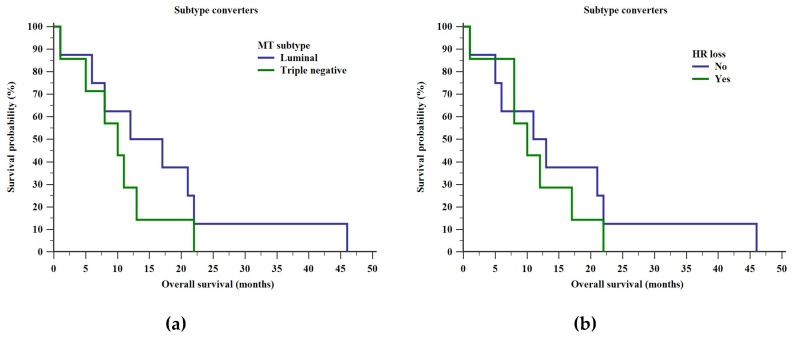
Additional factors influencing overall survival in subtype converters—(**a**) metastatic tumor (MT) subtype and (**b**) hormone receptor (HR) loss.

No differences in OSs between different MT subtypes in subtype converters were noted (log rank test, Chi square = 1.033, *p* = 0.309) as demonstrated in Figure 4. The same holds true when it comes to PT hormone receptor (HR) loss (log rank test, Chi square = 0.415, *p* = 0.520).

The proportion of CTC-positive patients decreased in the entire cohort between the baseline and follow-up—i+aCTC positive from 67.6 to 32.4%, aCTC positive from 47.1 to 20.6% and iCTC from 67.6 to 32.4% positive patients. In addition, the median i+aCTC, aCTC and iCTC counts decreased non-significantly after therapy—20 to 6 i+aCTC (*p* = 0.096); 4.5 to 1 aCTC (*p* = 0.8) and 15.5 to 3.5 iCTC (*p* = 0.24), respectively (Table 2).

In both the subtype converter and subtype stable subgroups the i+aCTC, aCTC and iCTC counts decreased on therapy but the reductions were not statistically significant, albeit more pronounced in the subtype converter subgroup (Table 2). Interestingly, median differences between initial and follow-up aCTC and iCTC counts showed a median increase in said counts only in the subtype converter subgroup. No statistically significant differences were observed in initial and follow-up i+aCTC, aCTC or iCTC counts between those experiencing tumor subtype conversion and those without it. However, subtype stable patients had achieved higher absolute counts in all categories of CTCs.

## 3. Discussion

Our data point towards a poorer overall survival of the intrinsic subtype converters (Figure 1). These observations are supported by several recent studies implicating hormone receptor dynamics as an independent prognostic factor of MBC at all stages of tumor progression [7,8,10,13,14,17,18]. In confirmation of previous studies, intrinsic subtype change occurred in a minority of patients (44.1%) during disease recurrence [20]. The majority of those converters changed to a more aggressive intrinsic subtype. This explains the diminution of OS in this group.

Shorter OSs in subtype converters compared to subtype stable patients did not seem to be due to the PT or MT intrinsic subtypes, or HR loss as demonstrated in Figure 3 and Figure 4. These findings are surprising given that multiple authors have shown that triple negative PTs have an unfavorable prognosis even if subtype conversion is not taken into account [7,8,13,21,22]. However, since a single patient had a triple negative subtype stable BC, it is not possible to draw any useful conclusions regarding the impact of PT subtype on OS in subtype stable patients. Similarly, a reduction in hormone receptor expression during a BC’s evolution through mutation has been associated with a worse prognosis in a number of studies [7,10,14,17,18,23,24]. As ESR1, PGR, ERBB2 and MKI67 reflect only a subset of prognostically relevant gene expressions, the general subtype change (weather to triple negative or to luminal subtypes) might reflect a general tendency towards mutations and therefore a more aggressive state [25,26,27,28].

We did not find any association between PFS and intrinsic subtype stability (Figure 1). Likewise, PFSs did not differ between patients with luminal and triple negative PTs in both the subtype converter and subtype stable subpopulations as shown in Figure 2. There does not seem to be any other comparable data published in this context thus far. However, our cohort is not big enough to ensure an adequately powered study. Thus, the differences discussed in this paragraph could well exist but remain undetected by an underpowered study such as ours.

We have noted higher total CTC, aCTC and iCTC counts in subtype converters compared with subtype stable patients in our study. Also, more substantial reductions in i+aCTCs, aCTCs and iCTCs were seen in subtype converters. However, these changes were not statistically significant. Other authors have observed higher total CTC counts in subtype converters [29,30]. CTCs are not a monolithic cell population, but a conglomerate of viable and apoptotic cells respectively defined as iCTCs and aCTC. Apoptotic CTCs seem to derive from therapy-induced apoptosis and apparently from spontaneous tumor apoptosis since there have also been detection in patients with no response to systemic therapy [31]. Nevertheless, patients with MBC had significantly lower numbers of aCTC compared to patients with early breast cancer [32].

We recognize that our study has several limitations. Firstly, the cohort size (34 patients) is small which correlates to a reduced study power which reflects in the fact that our study yielded several “null” results. However, we looked at events that are very rare even on a global scale. Furthermore, the study was very expensive, and the cohort inclusion criteria was extremely logistically and technically demanding, requiring the availability of biopsies of both primary and metastatic lesions with consecutive analysis of their mRNA profiles as well as enumeration of serum aCTCs, iCTCs and total CTCs for each patient in our cohort. We emphasize that the biomarker status of the primary tumor and the metastasis was analyzed by RT-qPCR and not the conventional immunohistochemistry adding to the scientific value of our study. Therefore, this cohort should be interpreted in the context of proof of principle.

As stated previously, our study yielded several “negative” results which might be perceived to be of lesser importance compared to studies with statistically significant results. On the contrary, our study can be interpreted in several ways that could inform clinical decision making and guide future research. If the observed differences in OS are not correlated to CTC counts or PT subtypes, subtype conversion itself could be the driving force behind unfavorable outcomes. In that respect, we would like to highlight that one of the implications of our study could be that in some cases of metastatic breast cancer the conversion of intrinsic subtype can have even a higher impact on survival than the count of apoptotic or intact CTCs emphasizing the importance of biopsies and re-biopsies of all available metastatic lesions resulting with potential earlier recognition of patients with poorer prognosis and in need of altered individualized therapy regimens. This is an insight with valuable practical consequences for clinical decision making and it is of utmost importance to us that it gets interpreted as our message to the community.

Further, even statistically nonsignificant results might reflect a clinically important real-world difference in a study as small as ours due to the suboptimal study power. Thus, one would be wise to study the data as a whole and not just the *p* values. For instance, therapeutic measures seem to have invariably led to a reduction in CTC counts with the effect more pronounced in subtype converters which is an interesting observation requiring further study. Additionally, subtype converters had higher baseline and final iCTC, aCTC and total CTC counts, possibly reflecting their worse prognosis. None of these observations were proven to represent a statistically significant difference between the groups and within the groups, but might be relevant none the less.

The limited cohort size compromised the power of our Kaplan-Meier analyses. Also, data on therapeutic interventions and comorbidities were not analyzed in our study. Further analysis on a bigger patient cohort is warranted in order to further elucidate the mechanisms behind the negative impact of intrinsic subtype conversion on overall survival.

Subgroup analyses according to the modality of systemic therapy were elaborated in Table 2 of our prequel paper [19]. The largest subgroup of the cohort had received chemotherapy (70.6%), followed by endocrine therapy (26.5%) and anti-HER2 therapy (2.9%). The CTC-positive counts rapidly decreased after one cycle of cytotoxic treatment, from 41.2% to 17.6% for the apoptotic CTC fraction and from 58.8% to 29.4% for intact CTCs. The comparably lower rates of CTCs in patients under endocrine treatment showed a decreasing trend from 8.8% to 2.9% for intact CTCs and from 5.9% to 2.9% for apoptotic CTCs [19].

## 4. Materials and Methods

This study enrolled patients treated for MBC at the Heidelberg University Hospital, Germany from April 2011 to May 2015. Both PT and MT biopsy specimens, as well as a blood sample obtained no more than 12 months since metastases were diagnosed, had to be available for a patient to be included in the study. The tissue samples were all formalin-fixed and paraffin-embedded and stored with corresponding blood samples at the tissue bank of the National Center for Tumor Diseases (NCT, Heidelberg, Germany). The study was approved by the ethics committee of the Medical Faculty Heidelberg of the Heidelberg University (approval no. S-295/2009, issued on 19 November 2009).

Each tissue sample was examined by a pathologist in order to confirm that it truly contains cancerous tissue. A single whole-face 10 μm-thick section of each tumor block was then subjected to processing via a RNA extraction kit (RNXtract^®^, BioNTech Diagnostics GmbH, Mainz, Germany). Having extracted a RNA sample we then analyzed it utilizing a commercial RT-qPCR kit (MammaTyper^®^, BioNTech Diagnostics GmbH) in an attempt to quantify the relative gene expression of ESR1, PGR, ERBB2 and MKI67 and two reference genes (B2M and CALM2) according to a pre-established protocol. Cut-offs for ERBB2, ESR1 and PGR were determined as described in our previous study [33]. We could then define the intrinsic tumor subtype using framework previously outlined by Goldhirsch et al.—luminal A, luminal B, HER 2 positive (also referred to as HER 2 enriched) and triple negative (TN) [21].

Total (i+aCTC), intact (iCTC) and apoptotic circulating tumor cell (aCTC) enumerations were performed on a peripheral blood sample at baseline and after cycle one of systemic therapy using CellSearch™ system (CellSearch™ Epithelial Cell Kit/CellSpotter™ Analyzer, Janssen Diagnostics, LLC, Raritan, NJ, USA) and the CellSearch™ assay (CellSearch™ Epithelial Cell Kit/CellSpotter™ Analyzer, Janssen Diagnostics, LLC). A ferrofluid coated with antibodies against EpCAM was used to separate epithelial cells which were subsequently labeled with the nuclear dye 4′,6-diamidino-2-phenylindole and immunostained with monoclonal antibodies specific for keratins and CD45. Trained observers using a semi-automated fluorescence-based microscopy system enumerated CTCs. Morphologically intact CTCs were designated iCTCs. The aCTCs were characterized by morphological criteria—disintegrated nuclei and/or a speckled pattern on keratin staining and/or M30 antibody staining. Patients were considered CTC positive if 5 or more CTCs per 7.5 mL were detected. Aside from the aforementioned methodology, researchers are working with microarrays of carbon nanotube surfaces as a new type of antigen-independent capture technique [34].

Demographic data and clinical characteristics were described as frequencies, percentages, means and standard deviations (normally distributed data), medians and ranges (non-normally distributed data). Progression free survival (PFS) and overall survival (OS) were compared between patients who experienced no intrinsic subtype conversion and those who did. We also compared PFSs and OSs between patients with luminal type, HER 2 positive and triple negative intrinsic subtype stable PTs. The identical analyses were conducted in a subpopulation of intrinsic subtype converters. The data was plotted and analyzed using Kaplan-Meier curves and the log rank test. Total CTC, aCTC and iCTC numbers and statuses were compared using the Chi-squared test, Sign test and the MWU test.

## 5. Conclusions

Intrinsic subtype conversion might be associated with a decreased overall survival rate. No other factors seem to unequivocally and statistically significantly influence overall survival in our study even though non-significantly decreased median overall survival was observed in patients with triple negative primary tumors. Total CTC, iCTC and aCTC counts were non-significantly higher in the subtype converters.

The hypothesis-generating nature of the findings from the present study emphasizes the importance performing biopsies and re-biopsies of all available metastatic lesions and mRNA-based monitoring of a possible intrinsic subtype shift paired with CTC-based liquid biopsies within the clinical decision-making process, resulting with earlier recognition of patients at risk and potentially leading to quicker optimization in individualized treatment of subtype-converted metastatic breast cancer patients.

## Figures and Tables

**Table 1:** Patient and tumor characteristics.

**Mean Age at PT Biopsy, Years, Mean ± SD**	52.3 ± 9.7
**Mean Age at MT Biopsy, Years, Mean ± SD**	58.1 ± 10.6
**Tumor Grade**	**Frequency (%)**
G1	0 (0%)
G2	18 (53%)
G3	12 (35%)
GX	4 (12%)
**Primary Tumor Intrinsic Subtype**	**Frequency (%)**
Luminal A	13 (38.2%)
Luminal B	13 (38.2%)
Triple-negative	7 (20.6%)
NA	1 (2.9%)
**Metastatic Tumor Intrinsic Subtype**	**Frequency (%)**
Luminal A	5 (14.7%)
Luminal B	14 (41.2%)
Triple-negative	8 (23.5%)
NA	7 (20.6%)
**Subtype Dynamics**	**Frequency (%)**
Subtype stable	11 (32.4%)
Subtype conversion	15 (44.1%)
NA	8 (23.5%)
**PFS, Months, Median (Range)**	6 (1–49)
**OS, Months, Median (Range)**	17 (1–52)

**Table 2:** Circulating tumor cells (CTC) dynamics.

CTC Counts	Subtype Stable	Subtype Converters	p_bg_	Total
i+aCTC1	10.65 (0–280)	25 (3–350)	0.11	20 (0–350)
i+aCTC2	3 (0–35)	9 (0–1140)	0.57	6 (0–1140)
p_wg_	1	0.11	/	0.096
∆ i+aCTC	0 (−280–19.7)	−5.5 (−263–1055)	0.79	−3 (−280–1055)
aCTC1	2.5 (0–200)	4 (0–170)	0.26	4.5 (0–200)
aCTC2	0 (0–23)	1 (0–250)	0.34	1 (0–250)
p_wg_	1	0.73	/	0.8
∆ aCTC	0 (−200–16)	−1 (−143–239)	0.74	0 (−200–239)
iCTC1	8.15 (0–80)	19 (0–180)	0.12	15.5 (0–180)
iCTC2	1 (0–15)	7 (0–890)	0.38	3.5 (0–890)
p_wg_	1	0.18	/	0.24
∆ iCTC	0 (−80–3.7)	−7 (−120–816)	0.74	−4.5 (−120–816)

∆ aCTC—difference in aCTCs; ∆ i+aCTC—difference in i+aCTCs; ∆ iCTC—difference in iCTCs; aCTC1—baseline aCTCs; aCTC2—follow-up aCTCs; i+aCTC1—baseline i+aCTCs; i+aCTC2—follow-up i+aCTCs; iCTC1—baseline iCTCs; iCTC2—follow-up iCTCs; p_wg_—statistical significance of the within group comparison; p_bg_—statistical significance of the between group comparison.
